# Repeat Transvenous Lead Extraction—Predictors, Effectiveness, Complications and Long-Term Prognostic Significance

**DOI:** 10.3390/ijerph192315602

**Published:** 2022-11-24

**Authors:** Andrzej Kutarski, Wojciech Jacheć, Dorota Nowosielecka, Marek Czajkowski, Łukasz Tułecki, Anna Polewczyk

**Affiliations:** 1 Department of Cardiology, Medical University, 20-059 Lublin, Poland; 2 2nd Department of Cardiology, Faculty of Medical Sciences in Zabrze, Medical University of Silesia, 41-800 Katowice, Poland; 3 Department of Cardiology, The Pope John Paul II Province Hospital, 22-400 Zamość, Poland; 4 Department of Cardiac Surgery, The Pope John Paul II Province Hospital, 22-400 Zamość, Poland; 5 Department of Cardiac Surgery, Medical University, 20-059 Lublin, Poland; 6 Institute of Medical Sciences, Jan Kochanowski University, 25-369 Kielce, Poland; 7 Department of Cardiac Surgery, Świętokrzyskie Center of Cardiology, 25-736 Kielce, Poland

**Keywords:** transvenous lead extraction, repeat extraction, safety, effectiveness, survival

## Abstract

Background: Data regarding repeat transvenous lead extraction (TLE) are scarce. The aim of study was to explore the frequency of repeat TLE, its safety, predisposing factors, as well as effectiveness of repeat procedures. Methods: Retrospective analysis of a large single-center database of 3654 TLEs. Results: Repeat TLE was a rare occurrence (193, i.e., 5,28% among 3654 TLEs). Subsequent re-extractions occurred in 12.21% of the patients. Lead failure was the most common cause of re-extraction (51.16%). Cox regression analysis showed that patients who were older at first implantation [HR = 0.987; *p* = 0.003], had infection-related TLE [HR = 0.392; *p* < 0.001] and complete procedural success [HR = 0.544; *p* = 0.034] were less likely to undergo repeat TLE. Functional leads left in place for continuous use [HR = 1.405; *p* = 0.012] or superfluous leads left in place (abandoned) [HR = 2.370; *p* = 0.011] were associated with an increased risk of undergoing a repeat procedure. Overall mortality in patients with repeat TLE and subsequent re-extraction in the entire FU period was similar to that in patients without a history of re-extraction [HR = 0.949; *p* = 0.480]. Conclusions: Repeat TLE was a rare occurrence (5.28%) among TLEs. Left of both active and nonactive leads during TLE increased the risk of re-extraction. Re-extraction has no effect on the long-term mortality.

## 1. Introduction

The number of patients with different types of cardiovascular implantable electronic devices (CIEDs) continues to grow, which is associated with a parallel increase in the long-term complications [1,2]. Transvenous lead extraction (TLE) is an important part of the long-term management of CIED recipients with lead-related problems and complications [3,4,5,6]. Indications for TLE, techniques, outcomes, complications and organizational aspects have been addressed in numerous reports from high-volume centers [7,8,9,10,11,12,13], data registries [14,15,16,17,18] and guidelines endorsed by cardiac societies [3,4,5,6]. However, from a lifespan perspective, younger/middle-aged groups of CIED patients are not free from future lead-associated problems. It means that old TLE centers (with a tradition of over a dozen or more years) will admit more and more patients with indications for repeat TLE. To the best of our knowledge, there are only a few papers addressing the problem of re-extraction [19,20,21,22]. Limited knowledge about the timing, causes, and short-, mid- and long-term effectiveness of repeat lead extraction prompted us to review our large database.

Goal of the study. The aim of this study was to determine the frequency, clinical scenarios (circumstances), safety as well as short-, mid- and long-term survival following repeat lead extraction among 3654 TLE procedures.

## 2. Methods

### 2.1. Study Population

All transvenous lead extraction procedures performed between March, 2006 and December, 2021 at a single high-volume center were screened. Patient clinical data, indications for TLE, CIED type and history of pacing, extracted leads, TLE complexity, efficacy and outcomes, the timing of repeat procedure and mortality during short-, mid- and long-term follow-up were retrospectively analyzed in our computerized database. The study population consisted of 3654 patients (38.2% female) ranging in age from 5 to 97 years, average 66.0 years.

### 2.2. Lead Extraction Procedure—Definitions

TLE indications, procedure effectiveness and complications were defined according to the latest TLE recommendations (2009 and 2017 HRS consensus and 2018 EHRA guidelines) [3,4,5,6]. The efficacy of TLE was expressed as the percentage of procedural success and clinical success. Procedural success was defined as the removal of all targeted leads and lead material from the vascular space with the absence of any permanently disabling complication or procedure-related death. Clinical success was defined as the removal of all targeted leads or retention of a small portion (<4 cm) of the lead that did not negatively impact the outcome goals of the procedure (i.e., residual lead did not increase the risk of perforation, embolic events, perpetuation of infection, or cause any undesired outcome) on condition of absence of any permanently disabling complication or procedure-related death [3,4,5,6]. The complications of TLE were also defined as major complications being those that were life threatening, resulted in significant or permanent disability or death, or required surgical intervention. Minor complications are undesired adverse events that require medical intervention, including minor procedural interventions, but do not significantly affect the patient’s function [3,4,5,6].

Procedure complexity was expressed as all lead extraction time (“sheath to sheath time”) and average time of single lead extraction (sheath-to sheath/number of extracted leads) and the necessity utility of second line tools and advanced tools [10,11,23,24,25,26,27].

Unexpected procedure difficulty so-called “technical problems” during TLE—situations which increased procedure complexity but not being complications. They were: break of extracted lead, loss of broken lead fragment—when main part of the lead was dilated and removed but remained free both endings, movable lead fragment which flowed usually info pulmonary vascular bed, block in lead venous entry/subclavian region block in lead venous entry preventing entry into the subclavian vein with a polypropylene catheter, Byrd dilator collapse/fracture, two leads strong scar connection, necessity to utilise other approach than lead venous entry and dislodgement of functional lead [10,11,24,25,26,27].

### 2.3. Procedure Information

Preoperative antibiotic prophylaxis was administered according to guidelines [23]. Standard stylets were most commonly used whereas locking stylets (Liberator Locking Stylet, Cook Medical Inc., Bloomington, IN, USA) were used for extraction of the oldest leads with a high estimated risk of fracture. Twisting and simple pulling were very rarely used due to the need to maintain or restore venous access in order to implant a new or temporary lead(s). Non-powered mechanical telescoping polypropylene sheaths (Byrd Dilator Sheaths, Cook Medical Inc., Bloomington, IN, USA) of all sizes and lengths were first-line tools for lead dissection and extraction. The second-line tools were powered mechanical sheath systems (Evolution Mechanical Dilator Sheath, Cook Medical Inc., Bloomington, IN, USA; TightRail Rotating Dilator Sheath, Phillips, Houston, TX, USA) or metal sheaths if the problem was located in implant vein and subclavian region. A combined approach, using two or more different (jugular, subclavian, femoral) access sites, was selected when conventional methods would be ineffective (lead proximal end in the cardiovascular space or in case of lead fracture). Laser and electrosurgical dissection sheaths were not used [10,24,25,26].

Over 17 years the organizational model of lead extraction evolved from procedures performed in the electrophysiology laboratory using intravenous analgesia/sedation [10] to procedures performed in the hybrid room in patients under general anesthesia. For the last 7 years, the core extraction team has consisted of the same highly experienced TLE operator, experienced echocardiographer and dedicated cardiac surgeon [24,25,26].

### 2.4. Dataset and Statistical Methods

The Shapiro–Wilk test showed that most continuous variables were normally distributed. For uniformity, all continuous variables are presented as the mean ± standard deviation. The categorical variables are presented as number and percentage.

**Figure 1:**
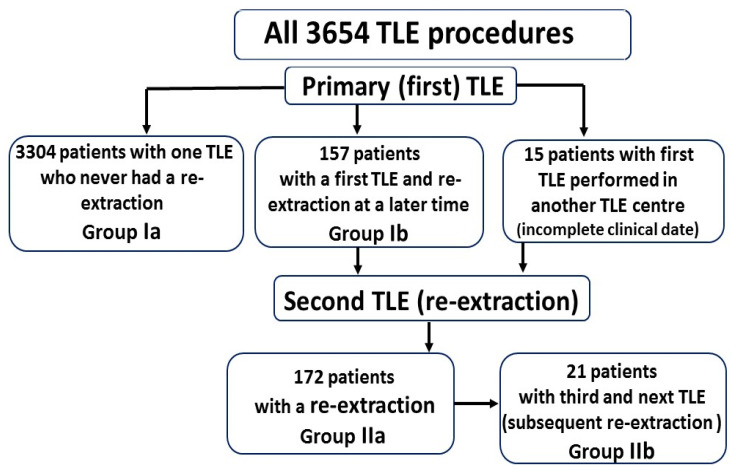
Group Ia: 3304 patients with a single TLE procedure, who have never had the re-extraction before. Group Ib: 157 patients with a first TLE procedure (analyzed at the moment of the procedure), in whom the re-extraction was performed later in the future. To analyze the differences and factors that can predict the necessity for the repetition of the TLE procedure we separated and compared group Ib from the Ia group. All patients (157) from group Ib had TLE procedures performed by the main operator, and the extraction and re-extraction data are available in the authors’ computer database. 15 patients were referred for a second TLE procedure (the first TLE procedure was performed in the other hospital). Moreover, taking into consideration the unique character of the re-TLE procedures we included those 15 patients in the re-extraction group called IIa (172 patients). Group IIa: 172 patients with a second TLE procedure (data analyzed at the moment of the re-extraction). Group IIb: 21 patients with a third and fourth TLE procedure.

Four subgroups were identified for comparative analysis. Group Ia: patients with s single TLE, 3304 patients. Group Ib: patients with a first TLE and re-extraction followed by repeat TLE at a later time, 157 patients. Group IIa: patients with a second TLE, 172 patients. Group IIb: patients with a third and fourth TLE, 21 patients (Figure 1). The statistical significance of the difference between groups (Ib, IIa, IIb vs. Ia) was tested using the nonparametric Pearson’s Chi^2^ test, Chi^2^ test with Yates correction or the unpaired Mann–Whitney U test, as appropriate. Univariate and multivariate Cox regression was used to identify the predictors of repeat TLE. Variables achieving statistical significance in comparison between groups Ia and Ib were included in the univariate model and next variables having a significant univariate test (*p* < 0.05) were selected for the multivariate analysis. Survival curves of patients split into groups according to the number of extraction procedures (Ia, IIa, IIb) were plotted. The log-rank test was performed to assess the differences in their course. A *p* value of less than 0.05 was considered as statistically significant. Statistical analysis was performed with Statistica version 13.3 (TIBCO Software Inc., Palo Alto, CA, USA).

### 2.5. Approval of the Bioethics Committee

All patients gave their informed written consent to undergo TLE and use anonymous data from their medical records, approved by the Bioethics Committee at the Regional Chamber of Physicians in Lublin no. 288/2018/KB/VII. The study was carried out in accordance with the ethical standards of the 1964 Declaration of Helsinki.

## 3. Results

Table 1 shows the annual number of repeat lead extractions for the last 16 years. The rate of re-extraction rose year-on-year from 2.60% to 21.00% (by 10.75 on average). It means that the percentage of re-extraction among all TLE procedures increased from 1.93% to 8.37%. Subsequent re-extractions occurred in 12.21% of cases after primary TLE (21 among 172).

Table 2, Table 3 and Table 4 summarize comparisons between the four patient subgroups. Table 2 contains information on 3654 patients including demographic and clinical data, indications for TLE and type of CIED. To find out whether there are any differences between an initial and a repeat extraction two pairs of groups were compared: patients with one TLE (group Ia) and patients with re-extraction (group IIa) as well as patients with one TLE (group Ia) and patients with a third TLE (group IIb). Patient-, indication- and CIED-related variables were compared to identify the risk factors for the need to perform subsequent TLEs. Additionally, patients with a second TLE (group IIa) are compared with patients undergoing a third or fourth TLE (group IIb).

### 3.1. General Differences between Groups of Patients with One TLE and Patients with re-Extraction

Compared with patients undergoing TLE only once (group Ia) those with re-extraction (group IIa) were younger both during TLE (60.76 vs. 66.65 years) and at first implantation (54.48 vs. 58.10), had less IHD (27.33 vs. 56.48%) and lower Charlson comorbidity index (3.79 vs. 4.81 points), more often lead dysfunction (29.65 vs. 21.85%), rarely pacemakers (57.6 vs. 71.13%) but more ICDs (30.81 vs. 21.01%) and CRT-D (10.47 vs. 6.91%)

### 3.2. Patient-Related Risk Factors for Repeat TLE

When comparing groups Ia and Ib, it was demonstrated that patients in group Ib were younger during TLE (57.77 vs. 66.65 years) and at first implantation (49.92 vs. 58.10), had less IHD (46.50 vs. 56.48%) and lower Charlson comorbidity index (3.57 vs. 4.81 points), more often mechanical lead damage with electrical failure (41.40 vs. 26.51%). Patients with third or fourth TLE did not differ significantly from group Ia except a higher rate of lead dysfunction.

Table 3 contains information on extracted leads, lead management strategy, TLE complexity, efficacy and outcomes of 3654 patients split into the same subgroups as in Table 2. This table analyses lead-related risk factors for procedure complexity, complications and effectiveness expressed as clinical success and procedural success. The fundamental goal is to answer the question whether a second lead extraction is more complicated, more difficult and riskier than an initial lead removal and whether procedure effectiveness (rates of clinical and procedural success) is comparable.

### 3.3. TLE Complexity, Efficacy and Outcomes in Patients with Single TLE and Those with Re-Extraction

Compared with patients undergoing TLE only once (group Ia) those with re-extraction (group IIa) had younger extracted leads (shorter dwell time of the oldest extracted lead and cumulative dwell time of extracted leads), but they had more CIED-related procedures before lead extraction, more abandoned leads, and more extractions of ICD and CS leads. The technical complexity of re-extraction was lower, which was in line with a shorter operative time. Re-extraction was also associated with lower rates of major complications, and no need for rescue cardiac surgery. Rates of clinical, procedural and partial radiographic success were similar.

### 3.4. Procedure-Related Risk Factors for Repeat TLE

Analysis of procedure-related risk factors in group Ia and group Ib showed that both subgroups were comparable in this aspect. Only patients in group Ib had less extracted leads and less often extraction of 3 or more leads during the first TLE. These patients also had slightly more minor complications and non-significantly less often required rescue operative intervention. Clinical success was comparable in both subgroups, but the rate of complete procedural success was lower in group Ib. In summary, the course of the first TLE did not indicate an increased risk of re-extraction.

### 3.5. Predictors of Repeat TLE, Results of Cox Regression Analysis

Univariable Cox regression analysis suggested that older patient age both at first implantation and during TLE, longer cumulative lead dwell time, more extracted leads, infectious indications for TLE and complete procedural success indicated a lower likelihood of repeat TLE. Mechanical lead defects, functional leads left in place for continued use or superfluous leads left in place may increase the likelihood of repeat lead extraction. Multivariable Cox regression analysis showed that the strongest predictors of repeat TLE were: older patient age at first implantation [HR = 0.987; *p* = 0.003], TLE due to infection [HR = 0.392; *p* < 0.001] and when complete procedural success of TLE was achieved [HR = 0.544; *p* = 0.034]. The likelihood of repeat TLE increased when functional leads were left in place for continuous use [HR = 1.405; *p* = 0.012] or superfluous leads were abandoned [HR = 2.370; *p* = 0.011] (Table 4).

### 3.6. Timing of Re-Extraction Procedure and Mortality after TLE

Table 5 presents two additional aspects of repeat lead extraction: the timing of re-extraction and short-, mid-, and long-time mortality after TLE among 3654 patients. The upper part of Table 5 gives information on the timing of repeat extraction after an initial procedure. Re-extraction was performed in 27.91% of patients within one year after TLE, in 42.44% within 2–5 years, and in 29.65% of patients within >5 years. Third or more extractions (rarely), which were necessary in 21 patients only, were performed in 33.33% within one year, in 52.34% within 2–5 years and in 14.29% of patients within >5 years. The bottom part of Table 5 presents short-, mid-, and long-time mortality after TLE. A total of 1192 (32.63%) patients died during a mean follow-up of 1948 days, with the highest rate in group Ia, but it cannot be ruled out that some of these patients would otherwise have undergone repeat TLE.

**Figure 2:**
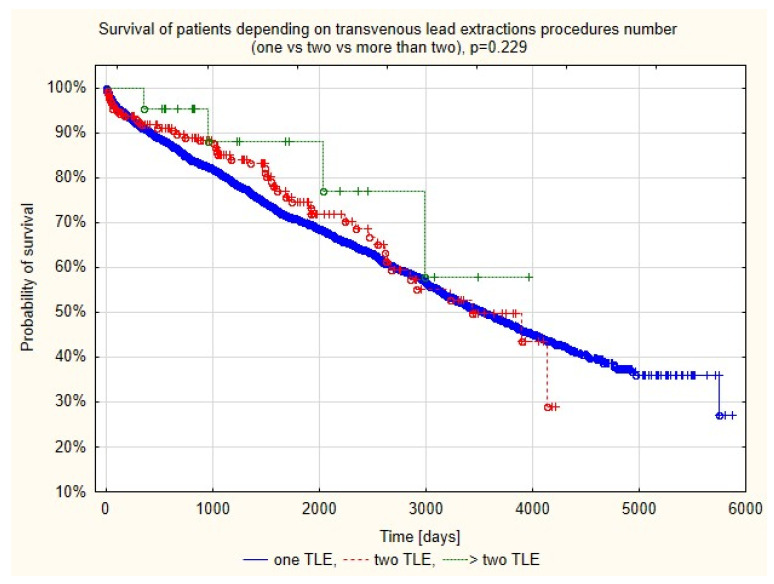
Kaplan–Meier survival curves depending on the number of TLE procedures: one (group Ia) vs. two (group IIa) vs. more than two (group IIb).

Cox regression analysis showed that mortality of patients with a second or more re-extractions in the entire FU period was similar to that in patients with a single TLE [HR = 0.949; 95% CI (0.822–1.097), *p* = 0.480]. This result was confirmed in log rank analysis of survival curves beginning the observation on the last TLE day. There were no statistically significant differences in their course (log rank *p* = 0.229), Figure 2.

One general conclusion is that mortality in the entire FU period was comparable between patients with two or more re-extraction procedures and those undergoing only one TLE.

### 3.7. Indications for Initial and Repeat TLE

Table 6 compares differences in indications for initial and repeat extractions in 172 patients (15 initial TLEs were performed in other centers). Taking into consideration the five main indication groups (systemic infection, local pocket infection, mechanical lead damage (with electrical lead failure), (non-damaged) lead dysfunction and other non-infectious indications we conclude that lead failure (mechanical lead damage with electrical failure) and lead dysfunction (in 44.77 and 24.42% of patients, respectively) are the most important causes of initial TLE. Infection and other causes of initial extraction were less common (14.53% and 16.28% of patients, respectively). The indications for re-extraction were varied. The percentage of systemic and local infections doubled (from 8.72 to 16.28% and from 5.81 to 11.63%), but the percentage of systemic infections was still lower compared to that in patients who did not undergo a second TLE. Other indications for re-extraction were adequately less common. Among 21 patients with subsequent re-extraction (third or fourth TLE) the pattern of indications appears to be similar.

### 3.8. Failure of New (Replaced) and Old Functional Leads Preserved during first TLE

Finally, a short analysis of lead failure (mechanical damage or lead dysfunction) was performed in all the 193 patients undergoing repeat extraction. If the initial extraction is performed for non-infectious reasons, only dysfunctional, superfluous leads can be removed or all leads can be replaced with new ones. In clinical practice, technical aspects play a decisive role (lead-to-lead adhesion or accidental damage to functional lead). We sought to answer the question whether it always makes sense to preserve a functional lead during lead extraction.

Table 7 shows that lead failure was the cause of lead re-extraction in 103 of the 193 repeat procedures (53.37%). Lead failure occurred in 88.35% of the new leads, but in 11.65% of the old (preserved) functional leads, which is 12/57 (21.05%) of all functional leads left in place during the first TLE. This result suggests that in the long-term perspective it is not always worthwhile to preserve functional leads (every fifth lead fails).

## 4. Discussion

Transvenous lead extraction plays a key role in the management of lead-related complications [3,4,5,6]. The effectiveness of the procedure is very high [7,8,9,10,11,12,13,14,15,16,17,18]. It appears useful in the management of CIED infections, replacement of failed leads, system upgrading, superfluous lead removal even in spite of varying degree of venous occlusion [27]. However, considering a long life perspective of young/middle-aged CIED recipients, limited lifetime of recently implanted leads, and a patient’s health likely to worsen after successful TLE, re-extraction for various reasons seems increasingly probable, especially in older high-volume centers. Our knowledge on lead re-extraction is limited to several papers [19,20,21,22].

Saeed O et al. demonstrated that of the 168 patients with a CIED extraction due to infection, nine patients (5.4%) underwent re-extraction due to a second infection during a mean follow-up of 4.4 years. Six re-extractions (67%) occurred in the first year, leading to an event rate of 3.9% within one year of reimplantation. Patients with re-extraction were younger, and pocket infection was the most common presentation of a second infection. The investigators suggest that further studies are warranted to identify predictors of recurring infection [19].

Fu H et al. reported on 38 patients who had undergone two or more lead extraction procedures compared to 439 patients who had a single procedure. The 5-year cumulative probability of a repeat procedure was 11%. Only 39% had device- and lead-related infections and the mean time from extraction to re-extraction was 63 months (5.5 years). In 72% of patients repeat extraction was performed for noninfectious indications (lead malfunction, abandoned lead, venous obstruction and other). Rates of major complications and procedural success were comparable to those in 439 patients who underwent single lead extraction. Higher NYHA class, noninfectious causes, and younger lead age were significant predictors of repeat lead extraction in univariate analyses; non-infection as the indication for lead extraction was the only independent predictor after adjustment for covariates [20].

Cay S et al. in their letter to editor pointed to a lack of experience with femoral approach during re-extraction, detailed procedural characteristics, the type of extraction (simple or complex). They suggested that evaluation of major complications and problems encountered during repeat extraction of ICD leads requires a larger sample size to increase the significance level of the findings [21].

Cha YM et al. made an attempt to clarify doubts but generally agreed that a much larger series of repeat TLE is needed to answer all questions [22].

Our results demonstrated an annual increase in the rate of re-extraction from 1.93% to 8.37% (10.75 per year on average). Lead failure/malfunction (mechanical lead damage or lead dysfunction) was the most common cause of re-extraction (51.16%). Infections and other non-infectious indications were less frequent (27.91% and 20.93%). However, the most remarkable result to emerge from the data comparison was the doubling rate of infections, reaching the values with respect to systemic infection observed in patients with a single extraction procedure. There were no significant system-related risk factors of undergoing re-extraction. However, older patient age at first CIED implantation, infectious indications for TLE, complete procedural success decreased the risk of repeat TLE. In contrast, the likelihood of re-extraction increased when active or superfluous leads were left in place (abandoned) during an initial TLE. These results are in part consistent with those of Saeed O [19] and Fu H et al. [20]. Re-extraction complexity and major complication rates were lower, but clinical and procedural success rates were similar to those observed during first TLE, which accords with the cited papers [19,20].

It is easy to explain lower complexity and lower rates of major complications during re-extraction. In 76.22% of patients undergoing initial extraction all leads were removed, and functional leads were left in place for continued use in only 22.99% of patients. Mean implant duration in patients with a single TLE and those with re-extraction was 93.45 months and 69.23 months, respectively. As we know, dwell time of extracted leads is the most important predictor of procedure complexity and major complications [7,8,9,10,11,12,13,14,15,16,17,18,19]. As we know, dwell time of extracted leads is the most important predictor of procedure complexity and major complications [7,8,9,10,11,12,13,14,15,16,17,18,19]. However, if remained risk factors of MC are generally known (female gender, young age, number of leads, abandoned leads, passive lead, low BMI), these are just a few studies that analyses the risk factors of increased complexity of the procedure. They are to be used to predict individual difficulty of the procedure: LED score (estimated time of fluoroscopy based on leads number, implant duration, ICD lead and vegetations) [28], MB score (need for advanced tools—based on implant duration, number of leads, passive lead and ICD lead) [29] and the Mazzone scale (need for advanced TLE techniques based on implant duration, number of leads and ICD lead) [30].

In spite of Cay S et al.’s hopes [21] there were no significant procedure-related risk factors for repeat extraction in our study. We can say that the course of the initial TLE in this large patient cohort does not indicate an increased risk associated with subsequent extraction procedures. Lead failure in many different forms was the reason for lead re-extraction in 103 of the 193 repeat procedures (53.37%). Re-extraction was performed late (1–5 years in 42.44%) and very late (over 5 years in 29.65%) after the initial extraction procedure. We demonstrated that repeat extraction had no influence on mortality in comparison to initial TLE.

The knowledge obtained from the TLE analyzes underpins the education in the field of CIED implantation and can be summarized: “when implanting the CIED, be mindful of possible future (safe) extractables” [3,4,5]. Repeated and subsequent re-TLE should result in knowledge useful for the lead extraction strategy. In all infectious cases, all leads are removed and there is no doubt [3,5]. However, we do not have any guidelines for handling still functional leads in non-infectious cases during TLE. Do we only replace the dysfunctional but necessary lead or, by the way, all the others. The recommendations leave the operators free to decide and recommend taking into account the age of the patient, the age of the leads, the risk of extraction the remaining leads, i.e., the risk/expected benefit balance in the future. Additionally, in this aspect, the results of our research seem very interesting. We have shown that functional lead left in place for continued use (HR 1.4122) and superfluous lead left in place (abandoned) (HR 2.358) (Table 4) were eminently significant risk factors for recurrent TLE. That is, by replacing all leads, we theoretically increase the risk of the current TLE, reducing the risk of disfunction/damage to an active but old lead and reducing the risk of MC during the next TLE because the implant duration will be shorter. In practice, in line with the general recommendations [3,5,6] in patients with a long life expectancy, we tend to opt for this option. The option of leaving old but functional leads is rather reserved for the elderly, with a shorter life expectancy. Further research into the optimal lead management funnel strategy is expected.

## 5. Conclusions

Repeat TLE was a rare occurrence (5.28%) among TLEs but the rate of re-extraction increased from 1.93% to 21.00% (by a mean of 10.75 annually). Subsequent re-extractions occurred in 12.21% of the patients undergoing the first repeat procedure. Lead failure was an important cause of re-extraction (51.16%), whereas infections and other non-infectious indications were less common (about 27.91 and 20.93%). Older patient age, infectious indications, complete procedural success of initial TLE decreased the likelihood of repeat extraction, in turn, leaving in place both active and nonactive leads increased the risk of re-extraction. Most re-extraction procedures were performed between 2–5 years (42.4%) and >5 years (29.6%) after the initial extraction. Overall mortality in the entire FU period in patients with re-extraction procedures was similar to that in patients with a single TLE.

### Study Limitations

This study has some limitations. It presents the experience of three centers but of the same first operator. The database was prospectively integrated, but analysis was performed retrospectively. About 70% of patients were referred for lead extraction from other districts in the country, and the reasons for performing initial and repeat extraction reflects general approach and not only the authors’ lead management strategy. We were not able to analyze in detail all re-infection cases after TLE because if all infected leads had been replaced, the patients returned to their local hospitals for continued care. Only those with preserved leads or patients with late infections were readmitted for lead extraction in one of our three centers. All procedures were performed using all types of mechanical systems but not laser powered sheaths. Finally, this paper describes the experience of a single high-volume operator. For this reason, it does not provide an overview of general TLE safety and efficacy in CIED patients with long lead dwell times.

## Figures and Tables

**Table 1:** Annual rates of repeat extractions in 5-year intervals.

Time Intervals [Years]	Number of TLEs N	First Re-Extraction N (%)	Re-Extraction Annually
2006–2010	672	13 (1.93%)	2.60
2011–2015	1337	50 (3.74%)	10.00
2016–2020	1394	88 (6.31%)	17.60
2021	251	21 (8.37%)	21.00
2006–2021	3654	172 (4.71%)	10.75
Subsequent extractions			
2006–2021	First re-extraction	Subsequent re-extraction (second or third)	Subsequent re-extraction annually
	172	21 (12.21%)	1.31

**Table 2:** Patient characteristics, indications for TLE and type of CIED.

	All TLE Procedures (Patients)	Patients with a Single TLE	Patients with a First TLE and Re-extraction at a Later Time	Patients with a Second TLE	Patients with a Third and Fourth TLE
	N = 3654 Average ± SD n (%)	N = 3304 Average ± SD n (%)	N = 157 Average ± SD n (%)	N = 172 Average ± SD n (%)	N = 21 Average ± SD n (%)
		Group Ia	Group Ib	Group IIa	Group IIb
Patient characteristics					
Patient age during TLE	65.98 ± 15.63	66.65 ± 15.01	57.77 ± 20.32 *p* < 0.001	60.76 ± 19.08 *p* < 0.001	63.43 ± 15.39 *p* = 0.299
Patient age at first implantation	57.58 ± 17.12	58.10 ± 16.56	49.92 ± 21.43 *p* < 0.001	54.48 ± 20.50 *p* = 0.115	57.86 ± 19.60 *p* = 0.744
Sex (female)	1395 (38.18)	1296 (39.23)	57 (36.31) *p* = 0.595	41 (23.84) *p* < 0.001	8 (38.09) *p* = 0.907
Etiology—ischemic heart disease	2033 (55.64)	1866 (56.48)	73 (46.50) *p* = 0.010	47 (27.33) *p* < 0.001	10 (47.62) *p* = 0.565
Renal failure (any)	751 (20.55)	692 (20.94)	19 (12.10) *p* = 0.009	38 (22.09) *p* = 0.367	2 (9.52) *p* = 0.257
Diabetes (any)	728 (19.92)	680 (20.36)	22 (14.01) *p* = 0.035	22 (12.79) *p* = 0.021	3 (14.29) *p* = 0.655
NYHA FC III or IV	548 (15.00)	502 (15.19)	49 (31.21) *p* = 0.010	26 (15.12) *p* = 0.486	2 (9.52) *p* = 0.381
LVEF < 40%	1126 (39.95)	1007 (30.71)	50 (31.85) *p* = 0.997	57 (35.63) *p* = 0.269	9 (42.86) *p* = 0.341
Congestive heart failure	673 (18.42)	616 (18.64)	16 (10.19) *p* = 0.018	35 (20.35) *p* = 0.480	6 (28.57) *p* = 0.352
Charlson comorbidity index	4.71 ± 3.67	4.81 ± 3.68	3.57 ± 3.45 *p* < 0.001	3.79 (3.41) *p* < 0.001	4.29 ± 3.59 *p* = 0.490
Indications for TLE Pearson’s Chi^2^ *p* < 0.001					
Systemic infection (with or without pocket infection)	793 (21.70)	747 (22.61)	15 (9.55) *p* < 0.001	28 (16.28) *p* < 0.001	3 (14.29) *p* = 0.490
Local (pocket) infection (*—including first TLE performed in other centers)	363 (9.93) * 366 (10.02)	335 (10.14)	7 (4.46) * 10 (6.37) *p* = 0.009	20 (11.63) *p* = 0.688	1 (4.76) *p* = 0.646
Mechanical lead damage (electrical failure) (*—including first TLE performed in other centers)	984 (26.93) * 996 (27.26)	876 (26.51)	65 (41.40) * 77 (49.04) *p* < 0.001	37 (21.51) *p* = 0.193	6 (28.57) *p* = 0.974
Lead dysfunction (exit/entry block, dislodgement, perforation, extracardiac pacing)	824 (22.55)	722 (21.85)	42 (26.75) *p* = 0.206	51 (29.65) *p* = 0.019	9 (42.86) *p* = 0.040
Abandoned lead/prevention of abandonment (upgrading, downgrading AF, multiple leads)	328 (8.98)	300 (9.08)	16 (10.19) *p* = 0.449	10 (5.81) *p* = 0.481	2 (9.52) *p* = 0.850
Threatening/potentially threatening lead (loops, free ends, left heart, LDTVD)	121 (3.31)	105 (3.18)	6 (3.82) *p* = 0.939	10 (5.81) *p* = 0.101	0 (0.00) *p* = 0.900
Other (MRI, cancer, painful pocket, pacing/ICD no longer needed)	109 (2.98)	99 (3.00)	2 (1.27) *p* = 0.561	8 (4.65) *p* = 0.343	0 (0.00) *p* = 0.842
Regained venous access (sympt. occlusion, SVC syndr., lead replacement/upgrading)	132 (3.61)	120 (3.63)	4 (2.55) *p* = 0.282	8 (4.65) *p* = 0.664	0 (0.00) *p* = 0.870
Type of CIED before TLE					
Device type—PM (any)	2565 (70.20)	2350 (71.13)	105 (66.88) *p* = 0.276	99 (57.56) *p* < 0.001	11 (52.38) *p* = 0.083
Device type—ICD	799 (21.87)	694 (21.01)	44 (28.03) *p* = 0.065	53 (30.81) *p* < 0.001	8 (38.10) *p* = 0.105
Device type—CRT-D	256 (7.01)	229 (6.91)	7 (4.46) *p* = 0.391	18 (10.47) *p* = 0.220	2 (9.52) *p* = 0.975
Only abandoned lead (unit removed earlier)	34 (0.93)	31 (0.94)	1 (0.64) *p* = 0.967	2 (1.63) *p* = 0.915	0 (0.00) *p* = 0.488

TLE—transvenous lead extraction, CIED—cardiac implantable electronic device, NYHA FC—New York Heart Association functional class, LVEF—left ventricular ejection fraction, AF—atrial fibrillation, LDTVD—lead-dependent tricuspid valve defect, MRI—magnetic resonance imaging, ICD—implantable cardioverter defibrillator, SVC—superior vena cava, PM—pacemaker, CRTD—cardiac resynchronization therapy defibrillator.

**Table 3:** TLE complexity, efficacy and outcomes.

	All TLE Procedures (Patients)	Patients with a Single TLE	Patients with a First TLE and Re-Extraction at a Later Time	Patients with a Second TLE	Patients with a Third and Fourth TLE
		Group Ia	Group Ib	Group IIa	Group IIb
	N = 3654 Average ± SD n (%)	N = 3304 Average ± SD n (%)	N = 157 Average ± SD n (%)	N = 172 Average ± SD n (%)	N = 21 Average ± SD n (%)
Extracted leads					
Dwell time of the oldest extracted lead [months]	99.69 ± 74.30	101.8 ± 74.74	93.45 ± 67.97 *p* = 0.096	69.23 ± 62.97 *p* < 0.001	61.24 ± 72.15 *p* = 0.002
Cumulative dwell time of extracted leads [years]	13.61 ± 12.58	14.02 ± 12.77	11.50 ± 10.36) *p* = 0.003	8.59 ± 9.30 *p* < 0.001	6.33 ± 6.74 *p* < 0.001
Number of CIED-related procedures before lead extraction	1.86 ± 1.08	1.83 ± 1.06	1.84 (1.12) *p* = 0.581	2.37 ± 1.12 *p* < 0.001	3.11 ± 1.84 *p* = 0.023
Extraction of abandoned lead (any)	378 (10.35)	348 (10.53)	19 (12.10) *p* = 0.497	10 (5.81) *p* = 0.087	1 (4.76) *p* = 0.721
Number of extracted leads per patient	1.65 ± 0.73	1.67 ± 0.73	1.48 ± 0.68 *p* < 0.001	1.54 ± 0.67 *p* = 0.030	1.33 ± 0.48 *p* = 0.058
Extraction of 3 and more leads	388 (10.62)	366 (11.08)	7 (4.46) *p* = 0.010	15 (8.72) *p* = 0.432	0 (0.00) *p* = 0.200
HV therapy (ICD) lead extraction	1001 (27.40)	881 (26.67)	50 (31.85) *p* = 0.223	64 (37.21) *p* = 0.002	6 (28.57) *p* = 0.964
CS lead extraction	233 (6.38)	205 (6.21)	6 (3.82) *p* = 0.986	21 (12.21) *p* = 0.060	1 (4.76) *p* = 0.455
Lead management strategy					
All leads were extracted (including abandoned leads)	2785 (76.22)	2557 (77.39)	96 (61.15) *p* < 0.001	121 (70.35) *p* = 0.037	11 (52.38) *p* = 0.015
Functional lead was left in place for continued use	840 (22.99)	723 (21.88)	57 (36.31) *p* < 0.001	50 (29.07) *p* = 0.110	10 (47.62) *p* = 0.032
Superfluous lead was left in place (abandoned)	20 (0.55)	16 (0.48)	4 (2.55) *p* = 0.019	0 (0.00) *p* = 0.480	0 (0.00) *p* = 0.905
Non-functional, superfluous lead was extracted	378 (10.35)	348 (10.53)	19 (12.10) *p* = 0.120	10 (5.814) *p* = 0.117	1 (4.76) *p* = 0.290
TLE complexity					
Procedural approach—left or right subclavian	3518 (96.28)	3185 (96.40)	148 (94.27) *p* = 0.244	164 (95.35) *p* = 0.612	21 (100.0) *p* = 0.767
Procedural approach—both sides	30 (0.82)	27 (0.82)	2 (1.27) *p* = 0.928	1 (0.58) *p* = 0.920	0 (0.00) *p* = 0.443
Procedural approach—other, combined	106 (2.90)	92 (2.79)	7 (4.46) *p* = 0.451	7 (4.07) *p* = 0.452	0 (0.00) *p* = 0.819
Technical problems during TLE (%)	733 (20.06)	672 (20.34)	35 (22.29) *p* = 0.945	24 (13.95) *p* = 0.052	2 (9.52) *p* = 0.170
2 or more technical problems (any)	158 (4.32)	148 (4.48)	4 (2.55) *p* = 0.532	6 (3.49) *p* = 0.866	0 (0.00) *p* = 0.857
Procedure duration (sheath-to-sheath time)	15.01 ± 22.76	15.15 ± 23.20	15.95 ± 18.66 *p* = 0.145	12.40 ± 18.13 *p* < 0.001	7.62 ± 9.09 *p* < 0.001
Procedure duration (average single lead extraction time)	8.91 ± 8.91	8.90 ± 12.53	10.26 ± 9.91 *p* < 0.001	8.10 ± 12.37 *p* < 0.001	6.36 ± 9.14 *p* < 0.001
TLE procedure efficacy and outcomes					
Minor complications (%)	267 (7.31)	240 (6.18)	16 (10.19) *p* = 0.389	11 (6.40) *p* = 0.950	4 (19.05) *p* = 0.913
Major complications (%)	73 (2.00)	69 (2.07)	3 (1.91) *p* = 0.928	1 (0.58) *p* = 0.272	0 (0.00) *p* = 0.915
Cardiac surgical intervention required (%)	43 (1.78)	42 (1.27)	1 (0.64) *p* = 0.770	0 (0.00) *p* = 0.259	0 (0.00) *p* = 0.645
Procedure-related death (intra-, post-procedural) (%)	6 (0.16)	6 (0.182)	0 (0.00) *p* = 0.687	0 (0.00) *p* = 0.681	0 (0.00) *p* = 0.845
Clinical success (%)	3576 (97.87)	3234 (97.88)	153 (97.45) *p* = 0.971	168 (97.67) *p* = 0.990	21 (100.0) *p* = 0.956
Complete procedural success (%)	3482 (95.29)	3153 (95.43)	143 (91.08) *p* = 0.013	166 (96.51) *p* = 0.747	20 (95.24) *p* = 0.615
Partial radiographic success (remained tip or < 4 cm lead fragment) (%)	144 (3.94)	128 (3.87)	10 (6.37) *p* = 0.010	5 (2.91) *p* = 0.688	1 (4.76) *p* = 0.683

TLE—transvenous lead extraction, HV—high voltage cardiac implantable cardioverter-defibrillator lead, ICD—implantable cardioverter-defibrillator, CS—coronary sinus.

**Table 4:** Predictors of repeat TLE, results of univariable and multivariable Cox regression analysis.

Cox Regression Model	Univariable Regression			Multivariable Regression		
	HR	95% CI	*p*	HR	95% CI	*p*
Patient age at first implantation [per year]	0.984	0.977–0.992	<0.001	0.987	0.978–0.995	0.002
Patient age during TLE [per year]	0.981	0.973–0.989	<0.001			
Charlson comorbidity index [per point]	0.956	0.910–1.005	0.079			
Etiology—ischemic heart disease	0.966	0.704–1.327	0.832			
Cumulative dwell time of extracted leads [per year]	0.982	0.967–0.998	0.025	0.985	0.965–1.006	0.153
Number of extracted leads per patient	0.614	0.477–0.791	<0.001	1.018	0.732–1.416	0.916
Renal failure (any)	0.868	0.541–1.393	0.558			
Diabetes (any)	0.814	0.518–1.278	0.371			
Congestive heart failure	1.079	0.646–1.804	0.770			
Infectious indications for TLE	0.281	0.179–0.442	<0.001	0.360	0.219–0.592	<0.001
Device type—ICD	1.533	1.083–2.171	0.016	1.405	0.962–2.050	0.078
Functional lead left in place for continued use	1.677	1.337–2.105	<0.001	1.412	1.085–1.836	0.010
Superfluous lead left in place (abandoned)	2.980	1.602–5.506	<0.001	2.358	1.195–4.653	0.013
All leads were extracted	0.480	0.349–0.661	<0.001			
Mean time of single lead extraction [per minute]	1.004	0.993–1.015	0.488			
Complete procedural success	0.552	0.324–0.940	0.029	0.531	0.302–0.933	0.028

TLE—transvenous lead extraction, ICD—implantable cardioverter-defibrillator.

**Table 5:** Timing of re-extraction procedure and short-, mid-, and long-time mortality after TLE.

	All TLE Procedures (Patients)	Patients with a Single TLE	Patients with a First TLE and Re-Extraction at a Later Time	Patients with a Second TLE	Patients with a Third and Fourth TLE
		Group Ia	Group Ib	Group IIa	Group IIb
	N = 3654 Average ± SD n (%)	N = 3304 Average ± SD n (%)	N = 157 Average ± SD n (%)	N = 172 Average ± SD n (%)	N = 21 Average ± SD n (%)
Timing of re-extraction					
Time since last CIED procedure (implantation, re-implantation, upgrading, downgrading, lead replacement)	45.44 ± 36.23	46.11 ± 47.76	47.76 ± 33.87 *p* = 0.345	29.74± 26.75 *p* < 0.001	14.27 ± 12.95 *p* < 0.001
No repeat TLE	3461 (94.72)	3304 (100.0)	157 (100.0)	0 (0.00)	0 (0.00)
Early re-extraction: 0–2 months	18 (0.49)	0 (0.00)	0 (0.00)	17 (9.88)	1 (4.76)
Delayed re-extraction: 3–12 months	37 (1.01)	0 (0.00)	0 (0.00)	31 (18.03)	6 (28.57)
Late re-extraction: 13–60 months	84 (2.30)	0 (0.00)	0 (0.00)	73 (42.44)	11 (52.38)
Very late re-extraction: > 60 months	54 (1.48)	0 (0.00)	0 (0.00)	51 (29.65)	3 (14.29)
All patients	3654 (100.0)	3304 (100.0)	157 (100.0)	172 (100.0)	21 (100.0)
Time since last extraction (months)	42.56 ± 40.30	0.00	0.00	44.03 ± 41.05)	30.57 (31.85)
Short-, mid- and long-term prognosis after TLE					
Follow-up [days] (min. ÷ max.)	1948 ± 1381 (1÷5874)	1927 ± 1375 (1 ÷ 5874)	1507 ± 1109 (9 ÷ 4181) *p* < 0.001	1537 ± 1152 (9 ÷ 4222) *p* < 0.001	1643 ± 1081 (353 ÷ 3962) *p* = 0.472
Alive during follow-up	2462 (67.38)	2198 (66.53)	117 (74.52)	130 (75.58)	17 (80.95)
Died during follow-up	1192 (32.63)	1106 (33.48)	40 (25.48)	42 (24.42)	4 (19.05)
Log-rank *p* = 0.229 (Ia vs. IIa vs. IIb)					
2-day mortality (first 48 h)	11	11	0	0	0
1-month mortality (2–30 days)	45	41	0	4	0
1-year mortality (31–365 days)	237	224	2	10	1
3-year mortality (366–1095 days)	300	282	8	9	1
>3-year mortality (> 1095 days)	599	548	30	19	2

TLE—transvenous lead extraction, CIED—cardiac implantable electronic devices.

**Table 6:** First time and repeated TLE indications.

TLE Indications	Systemic Infection	Local Pocket Infection	Mechanical Lead Damage	Lead Dysfunction	Other TLE Indications	All TLE n (%)
Indications for primary TLE						
Systemic infection	15	-	-	-	-	15 (8.72)
Local pocket infection	-	10	-	-	-	10 (5.81)
Mechanical lead damage	-	-	77	-	-	77 (44.77)
Lead dysfunction	-	-	-	42	-	42 (24.42)
Other TLE indications	-	-	-	-	28	28 (16.28)
All	15	10	77	42	28	172 (100.0)
Indications for repeat TLE in patients grouped depending on primary TLE indications						
Systemic infection	6	6	9	3	4	28 (16.28)
Local pocket infection	1	1	9	7	2	20 (11.63)
Mechanical lead damage	2	1	26	4	4	37 (21.51)
Lead dysfunction	4	1	14	23	9	51 (29.65)
Other TLE indications	2	1	19	5	9	36 (20.93)
All	15	10	77	42	28	172 (100.0)
Indications for repeat (third or fourth) TLE in patients, grouped depending on primary TLE indications						
Systemic infection	1	1	0	1	0	3 (14.29)
Local pocket infection	1	0	0	0	0	1 (4.76)
Mechanical lead damage	1	0	6	0	1	8 (30.10)
Lead dysfunction	0	0	0	7	0	7 (33.33)
Other TLE indications	0	0	0	1	1	2 (9.52)
All	3	1	6	9	2	21 (100.0)

TLE—transvenous lead extraction.

**Table 7:** Lead failure among the new (replaced) and old but functional saved during first TLE leads.

Lead Failure on Re-Extraction (all) 193 TLE Procedures		
	Attached Lead New/Old	Number of Cases
Lead dysfunction	new	50 (48.54%)
Mechanical lead damage	new	41 (39.54%)
Lead dysfunction	old	9(8.74%)
Mechanical lead damage	old	3 (2.91%)
All		103 (100%)

## Data Availability

Readers can access data supporting the conclusions of the study upon reasoned request to the authors.
